# Comprehensive analysis of ceRNA networks reveals prognostic lncRNAs related to immune infiltration in colorectal cancer

**DOI:** 10.1186/s12885-021-07995-2

**Published:** 2021-03-09

**Authors:** Jingyi Chen, Yuxuan Song, Mei Li, Yu Zhang, Tingru Lin, Jie Sun, Di Wang, Yulan Liu, Jingzhu Guo, Weidong Yu

**Affiliations:** 1 Department of Central Laboratory & Institute of Clinical Molecular Biology, Peking University People’s Hospital, Beijing, 100044 China; 2 Department of Gastroenterology, Peking University People’s Hospital, Beijing, 100044 China; 3 Department of Urology, Tianjin Medical University General Hospital, Tianjin, 300052 China; 4 Department of Paediatrics, Peking University People’s Hospital, Beijing, 100044 China

**Keywords:** ceRNA network, lncRNA, Immune infiltration, Prognosis, CRC

## Abstract

**Background:**

Competing endogenous RNA (ceRNA) represents a class of RNAs (e.g., long noncoding RNAs [lncRNAs]) with microRNA (miRNA) binding sites, which can competitively bind miRNA and inhibit its regulation of target genes. Increasing evidence has underscored the involvement of dysregulated ceRNA networks in the occurrence and progression of colorectal cancer (CRC). The purpose of this study was to construct a ceRNA network related to the prognosis of CRC and further explore the potential mechanisms that affect this prognosis.

**Methods:**

RNA-Seq and miRNA-Seq data from The Cancer Genome Atlas (TCGA) were used to identify differentially expressed lncRNAs (DElncRNAs), microRNAs (DEmiRNAs), and mRNAs (DEmRNAs), and a prognosis-related ceRNA network was constructed based on DElncRNA survival analysis. Subsequently, pathway enrichment, Pearson correlation, and Gene Set Enrichment Analysis (GSEA) were performed to determine the function of the genes in the ceRNA network. Gene Expression Profiling Interactive Analysis (GEPIA) and immunohistochemistry (IHC) were also used to validate differential gene expression. Finally, the correlation between lncRNA and immune cell infiltration in the tumor microenvironment was evaluated based on the CIBERSORT algorithm.

**Results:**

A prognostic ceRNA network was constructed with eleven key survival-related DElncRNAs (MIR4435-2HG, NKILA, AFAP1-AS1, ELFN1-AS1, AC005520.2, AC245884.8, AL354836.1, AL355987.4, AL591845.1, LINC02038, and AC104823.1), 54 DEmiRNAs, and 308 DEmRNAs. The MIR4435-2HG- and ELFN1-AS1-associated ceRNA subnetworks affected and regulated the expression of the COL5A2, LOX, OSBPL3, PLAU, VCAN, SRM, and E2F1 target genes and were found to be related to prognosis and tumor-infiltrating immune cell types.

**Conclusions:**

MIR4435-2HG and ELFN1-AS1 are associated with prognosis and tumor-infiltrating immune cell types and could represent potential prognostic biomarkers or therapeutic targets in colorectal carcinoma.

**Supplementary Information:**

The online version contains supplementary material available at 10.1186/s12885-021-07995-2.

## Background

Colorectal cancer (CRC), the third most common cancer and the second leading cause of cancer mortality worldwide, is caused by the accumulation of long-term genetic mutations and epigenetic alterations [1]. Although recent interventional and therapeutic advances have greatly improved the overall survival (OS) of CRC, patients with advanced CRC still show poor prognosis due to the high frequency of recurrence and metastasis [2]. Investigation of the molecular mechanisms underlying CRC, from initiation to metastasis, is urgently needed to improve its early diagnosis, treatment, and prognosis. Long noncoding RNAs (lncRNAs) are noncoding transcripts that range from 200 to 100,000 nucleotides in length and lack protein-coding capacity. They function as signals, decoys, guides, and scaffolds in gene regulatory networks [3]. The regulatory mechanism of lncRNA depends on its subcellular localization. Cytoplasmic lncRNAs primarily participate in post-transcriptional and translational regulation by acting as microRNA (miRNA) sponges and modulating mRNA stability. An increasing number of lncRNAs have been found to participate in CRC initiation and progression by functioning as competing endogenous RNAs (ceRNAs) [4–6]. Specifically, lncRNAs derepress target gene expression by competitively binding to shared sequences of miRNAs [7, 8]. Recent studies showed that ceRNA crosstalk was involved in various biological processes, including CRC cell proliferation, invasion, epithelial-to-mesenchymal transition (EMT), metastasis, and immune infiltration [9–13].

**Fig. 1:**
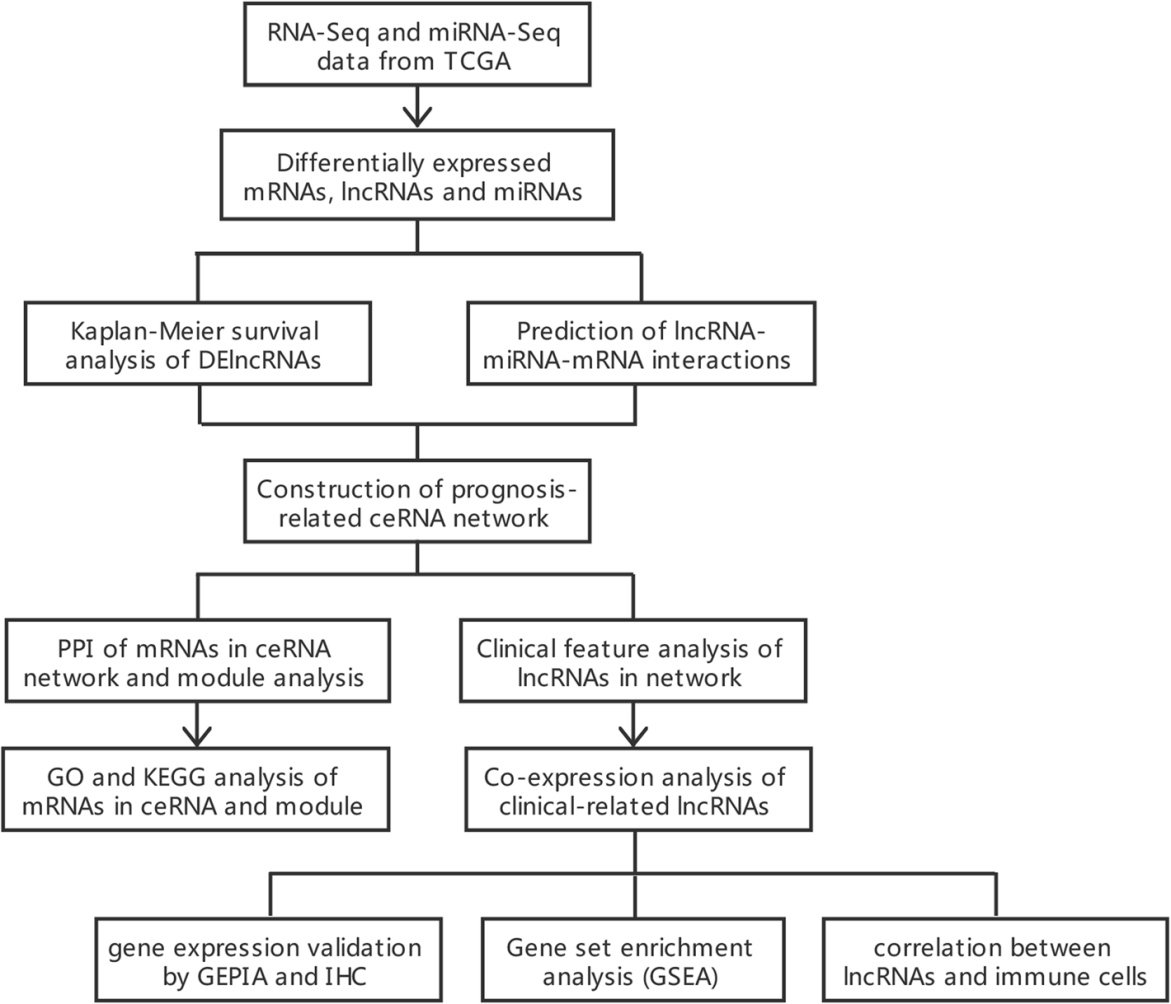
Flowchart for the identification and analysis of prognostic lncRNAs in the ceRNA network

In the present study, we acquired the expression profiles and clinical information for colon adenocarcinoma (COAD) and rectal adenocarcinoma (READ) from The Cancer Genome Atlas (TCGA). Differentially expressed mRNAs (DEmRNAs), lncRNAs (DElncRNAs), and miRNAs (DEmiRNAs) were then identified by comparing tumor samples and normal samples. We performed survival analysis and predicted RNA interaction pairs to construct a prognosis-related ceRNA network. The protein–protein interaction (PPI) network was embodied in Cytoscape to identify module clusters and hub genes. Gene Ontology (GO) and Kyoto Encyclopedia of Genes and Genomes (KEGG) analysis were performed to explore the biological functions of the mRNAs in the ceRNA and module network. In addition, we analyzed the correlations between key lncRNAs and the clinical features of CRC patients. Co-expression analysis between lncRNAs and target genes was performed to verify the ceRNA hypothesis. Eventually, we established ceRNA regulatory subnetworks based on the above results. GEPIA and immunohistochemistry were used to validate the differential expression of genes, whereas GSEA was used to predict the underlying functions of the genes in the subnetwork. The CIBERSORT algorithm was used to estimate the proportion of immune cells for further correlation analysis (Fig. 1).

## Methods

### Data downloading and pre-processing

The mRNA, lncRNA, and miRNA expression profiles of the CRC patients were downloaded from TCGA database (https://cancergenome.nih.gov/). We collected RNA-Seq data for 612 samples (568 CRC and 44 normal samples) in HTSeq-Counts files and miRNA-Seq data for 548 samples (539 CRC and nine normal samples). The sequence data were derived from Illumina HiSeq RNASeq and miRNASeq platforms, which were publicly available. GENCODE (https://www.gencodegenes.org/) was used to convert the RNA-Seq data into lncRNAs (sense_overlapping, lincRNA, 3prime_overlapping_ncRNA, processed_transcript, antisense, and sense_intronic) and mRNAs (protein-coding).

### Differential expression analysis of RNAs

To screen differentially expressed RNAs (DERNAs) between tumor samples and normal samples, we used the “edgeR” package in R software to standardize and analyze the downloaded data. DEmRNAs, DElncRNAs, and DEmiRNAs were identified with the criteria of |log2 fold change (FC)| > 1.0 and adjusted *P* value < 0.05. Heatmap clustering and volcano plot were generated using R package “edgeR” to reveal the differential expressions of the DEmRNAs, DElncRNAs, and DEmiRNAs.

### Prediction of RNA interaction pairs

The online tool DIANA-LncBase version 3 (http://www.microrna.gr/LncBase) [14] was used to predict the potential miRNAs targeted by DElncRNAs and determine lncRNA–miRNA interaction pairs. MiRDB (http://www.mirdb.org/) [15], miRTarBase (http://mirtarbase.mbc.nctu.edu.tw/) [16], and TargetScan (http://www.targetscan.org/) [17] databases were used to predict the target genes of DEmiRNAs and miRNA–mRNA interaction pairs. The R package “VennDiagram” was used to compare the target genes with the DEmRNAs to obtain overlapping genes.

### Survival analysis and ceRNA network construction

RNA expression profiles and related clinical information of the CRC patients were acquired from TCGA database. To identify lncRNAs significantly associated with patient prognosis, we used the R package “survival” to perform Kaplan-Meier curve analysis of DElncRNAs. DElncRNAs with *P* < 0.05 were considered to be key lncRNAs for construction of the next network. Based on the hypothesis that lncRNA acts as a miRNA sponge to regulate mRNA expression [7], we selected key lncRNAs and lncRNA–miRNA–mRNA interaction pairs to build the ceRNA regulatory network. Ultimately, we combined specific lncRNA–miRNA and miRNA–mRNA pairs to establish a prognosis-related network. The established ceRNA network was visualized using Cytoscape v3.6.0 software [18]. Univariate and multivariate Cox regression analyses were performed to identify the independent prognostic indicators for overall survival (OS).

### Construction of PPI network

To better understand the interactions of DEmRNAs in the ceRNA network, we constructed a PPI network using the Search Tool for the Retrieval of Interacting Genes (STRING; https://string-db.org/) [19] database. The median confidence score (0.4) was used to filter protein interactions. Cytoscape 3.6.0 was used to embody the constructed PPI networks. In addition, we used the plugin cytoHubba to identify hub genes in the PPI network according to degree of connectivity. The top 30 hub genes were identified through the Degree ranking method. The MCODE plugin was used to analyze the PPI network and select the top module for subnetwork analysis.

### Functional annotation analysis

To explore the potential biological functions and pathways of mRNAs in the ceRNA and module network, we performed Gene Ontology (GO) and Kyoto Encyclopedia of Genes and Genomes (KEGG) pathway analysis through the R package “ClusterProfiler”. GO terms and KEGG pathways with *P* < 0.05 were considered significantly enriched.

### Clinical feature analysis of key lncRNAs

Clinical information, including age, sex, tumor stage, and TNM stage, was extracted from TCGA database. Based on the prognosis-related ceRNA network, we evaluated the associations between key lncRNAs and the clinical features of CRC patients using the R package “ggpubr”.

### Correlation analysis of lncRNAs and mRNAs

We performed Pearson correlation analysis and selected the positive correlations between the lncRNAs and mRNAs in ceRNA network. Pearson correlation coefficient > 0.4 and *P* < 0.001 were used as inclusion criteria. Based on the above co-expression relationships, we constructed a ceRNA subnetwork to investigate the potential mechanisms.

### Validation of gene expression

Gene Expression Profiling Interactive Analysis (GEPIA; http://gepia.cancer-pku.cn/) is an online analysis tool based on TCGA and the Genotype-Tissue Expression (GTEx) dataset [20]. Differential expression boxplot in GEPIA was used to compare the expression levels of genes between tumor samples and normal samples. The Human Protein Atlas (THPA; https://www.proteinatlas.org) is a database that provides immunohistochemistry-based maps of human protein profiles for cancer tissues and normal tissues and cells [21]. We obtained immunohistochemistry images and data from this database to validate gene expression at the protein level. Staining intensity was evaluated as follows: no staining, 0; low staining, 1; medium staining, 2; high staining, 3. Staining quantity was scored as follows: none, 0; < 25%, 1; 25–50%, 2; 51–75%, 3; > 75%, 4. The total staining score was calculated by multiplying these two scores. Differences in the total staining scores between normal and CRC tissues were evaluated using the unpaired t-test. *P* < 0.05 was considered statistically significant.

### Gene set enrichment analysis

To explore the biological pathways of genes in a subnetwork, Gene Set Enrichment Analysis (GSEA) analysis was performed based on TCGA dataset. According to the cutoff value, the samples were divided into high and low expression phenotypes. The gene set permutations were performed 1000 times per analysis. Gene sets with |normalized enrichment score (NES)| > 1 and P < 0.05 were considered significantly enriched.

### Evaluation of immune cell infiltration

Normalized gene expression data were uploaded to TIMER2.0 (http://timer.cistrome.org/) [22], a website that provides comprehensive evaluation and analysis of tumor-infiltrating immune cells, to estimate the relative abundance of 22 immune cell types based on the CIBERSORT algorithm. To evaluate the correlation between lncRNA and immune cell infiltration in the tumor microenvironment, CRC samples from the TCGA database were divided into high and low lncRNA expression groups. The Wilcoxon rank-sum test was performed to compare the differences in immune cell infiltration between the high and low lncRNA expression groups.

## Results

### Differentially expressed lncRNAs, miRNAs, and mRNAs

**Fig. 2:**
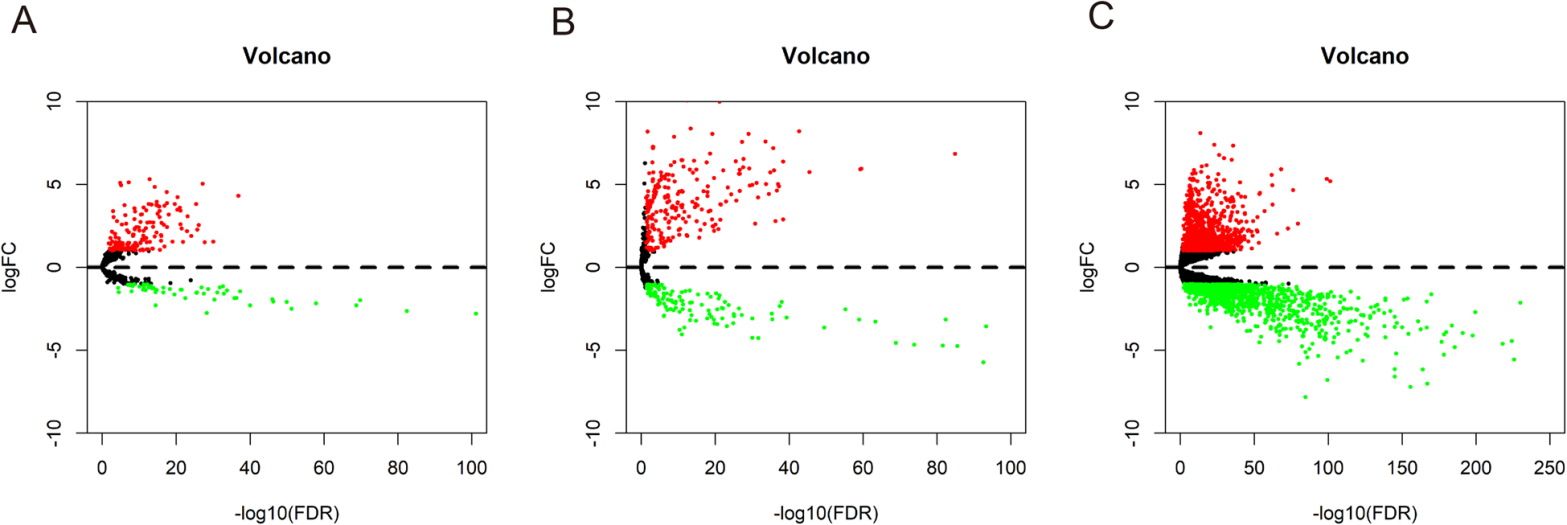
Volcano plots of differentially expressed RNAs. **a** Volcano plot of lncRNAs. **b** Volcano plot of miRNAs. **c** Volcano plot of mRNAs. The red dots represent upregulated RNAs, and the green dots represent downregulated RNAs

We obtained the expression profiles of 19,645 mRNAs, 14,166 lncRNAs, and 1881 miRNAs from TCGA database. A total of 2480 DEmRNAs, 217 DElncRNAs, and 376 DEmiRNAs were identified with the criteria of |log2 FC| > 1 and adjusted *P* < 0.05. Among them, 1325 DEmRNAs, 159 DElncRNAs, and 243 DEmiRNAs were upregulated, whereas 1155 DEmRNAs, 58 DElncRNAs, and 133 DEmiRNAs were downregulated (Supplementary Figure S1 and Fig. 2).

### Prediction of lncRNA–miRNA and miRNA–mRNA interactions

We predicted lncRNA–miRNA interaction pairs based on DElncRNAs using the online tool DIANA-LncBase version 3 and found that 153 of the 217 DElncRNAs might target 199 of the 376 DEmiRNAs. Subsequently, miRTarBase, miRDB, and TargetScan databases were used to identify miRNA–mRNA interaction pairs. Target mRNAs that were recognized by all three databases were selected as candidate genes. The results showed that 184 of the 199 specific DEmiRNAs might target 3475 candidate genes. After comparing the 3475 candidate genes with 2480 DEmRNAs, we obtained 455 overlapping genes and 1148 miRNA–mRNA interaction pairs.

### Construction of prognosis-related ceRNA network

**Fig. 3:**
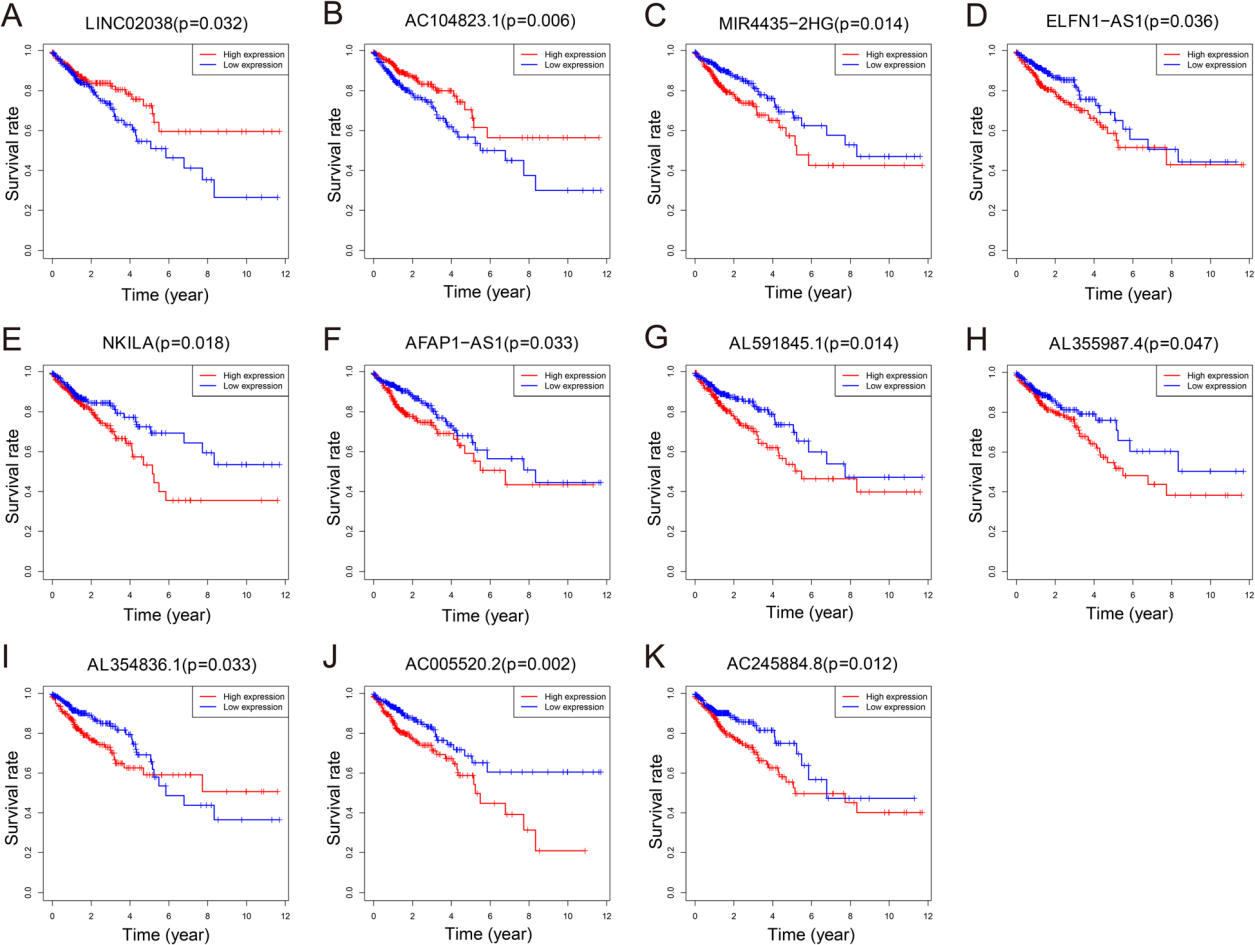
Kaplan-Meier survival curves of eleven key lncRNAs related to overall survival (OS). LINC02038 (**a**) and AC104823.1 (**b**) were positively correlated with OS. MIR4435-2HG (**c**), ELFN1-AS1 (**d**), NKILA (**e**), AFAP1-AS1 (**f**), AL591845.1 (**g**), AL355987.4 (**h**), AL354836.1 (**i**), AC005520.2 (**j**), and AC245884.8 (**k**) were negatively correlated with OS. The red lines represent the high expression groups, and the blue lines represent the low expression groups

A ceRNA network was constructed using 2489 lncRNA-miRNA pairs and 1148 miRNA-mRNA pairs using Cytoscape software. The association of 153 lncRNAs in the ceRNA network with patient prognosis was analyzed using Kaplan-Meier survival curves. The results showed that 11 of the 153 lncRNAs were significantly associated with OS. The 11 prognosis-related DElncRNAs were considered key lncRNAs. LINC02038 and AC104823.1 were positively correlated with OS, whereas MIR4435-2HG, NKILA, AFAP1-AS1, ELFN1-AS1, AC005520.2, AC245884.8, AL354836.1, AL355987.4, and AL591845.1 were negatively correlated with OS (Fig. 3). Ultimately, we constructed a survival-related ceRNA network with 11 key lncRNAs, 54 DEmiRNAs, and 308 DEmRNAs. This network contained 373 nodes and 739 edges (Supplementary Figure S2). A total of 11 lncRNAs and 308 mRNAs in the ceRNA network were included in the univariate cox regression analysis. Six lncRNAs and 28 mRNAs with *P* < 0.05 were subjected to multivariate COX regression analysis. The lncRNA ELFN1-AS1 and the SPTBN2, CCNF, and VEGFA mRNAs were identified as independent prognostic factors for OS in CRC patients (Supplementary Table S3).

### PPI network and module analysis

**Fig. 4:**
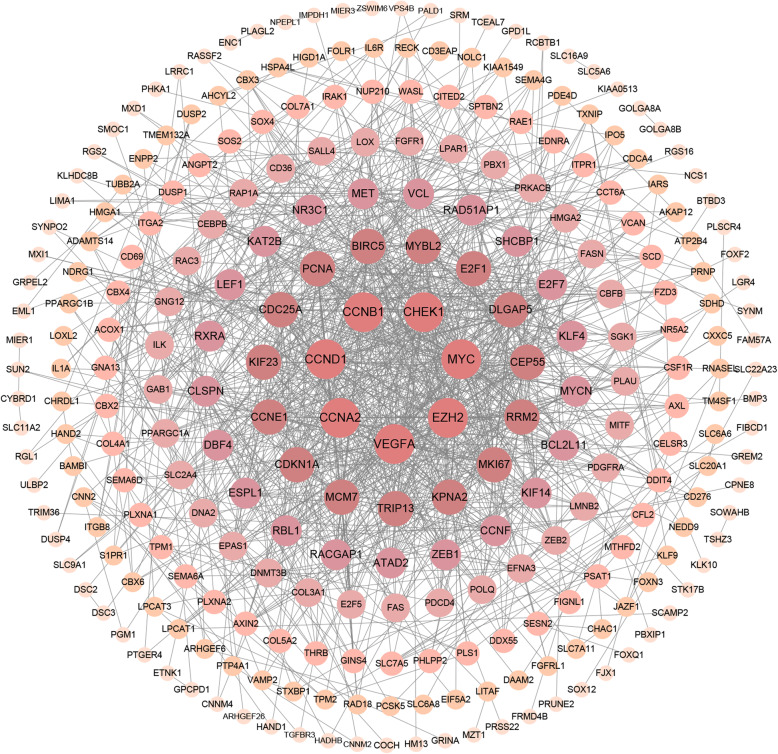
The protein–protein interaction (PPI) network of differentially expressed genes in the prognosis-related ceRNA network. Each node corresponds to a protein-coding gene, and the size of the node represents the connection degree

**Fig. 5:**
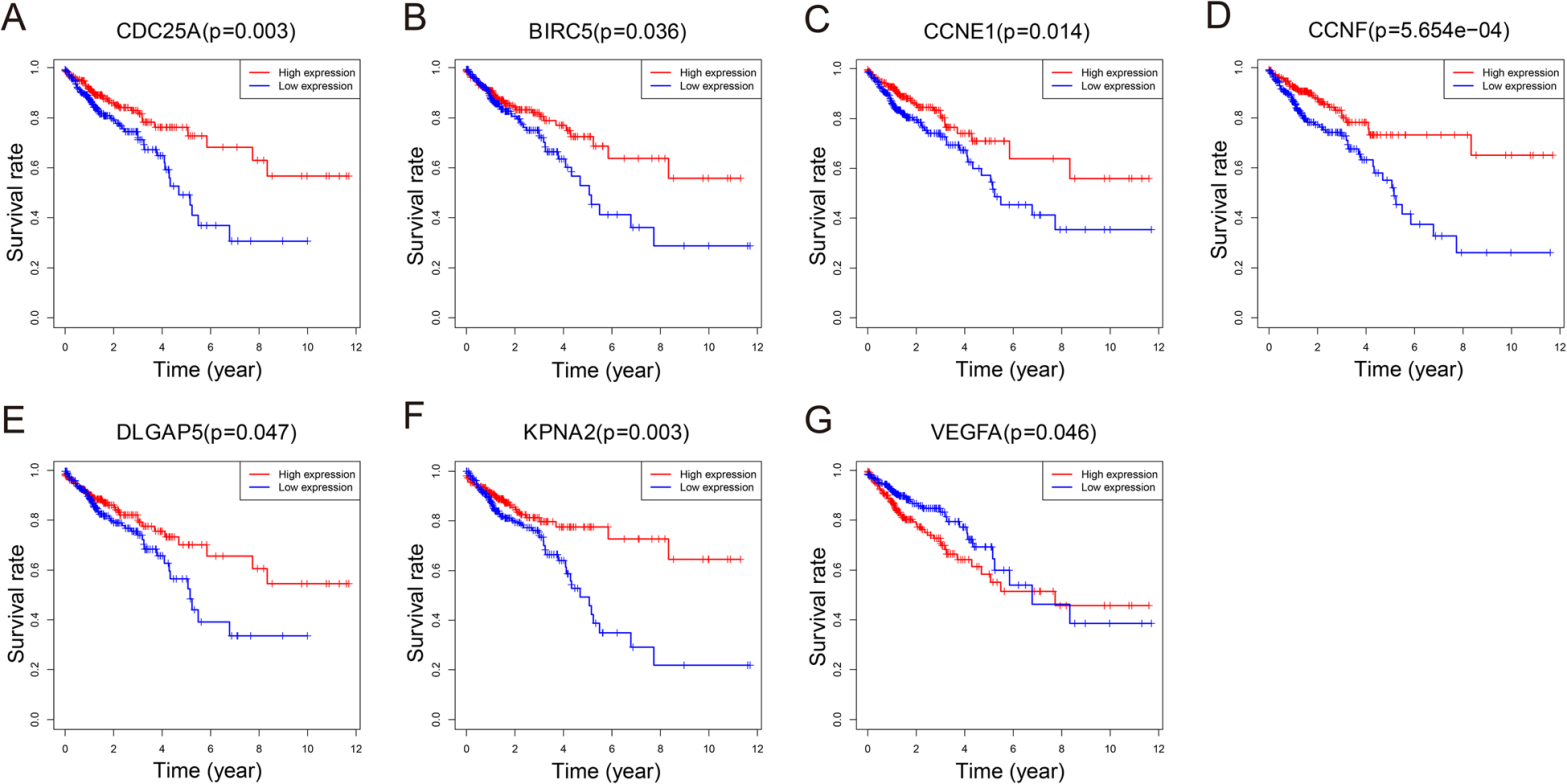
Kaplan-Meier survival curves of seven hub genes related to overall survival (OS). CDC25A (**a**), BIRC5 (**b**), CCNE1 (**c**), CCNF (**d**), DLGAP5 (**e**), and KPNA2 (**f**) were positively correlated with OS, and VEGFA (**g**) was negatively correlated with OS. The red lines represent the high expression groups, and the blue lines represent the low expression groups

**Fig. 6:**
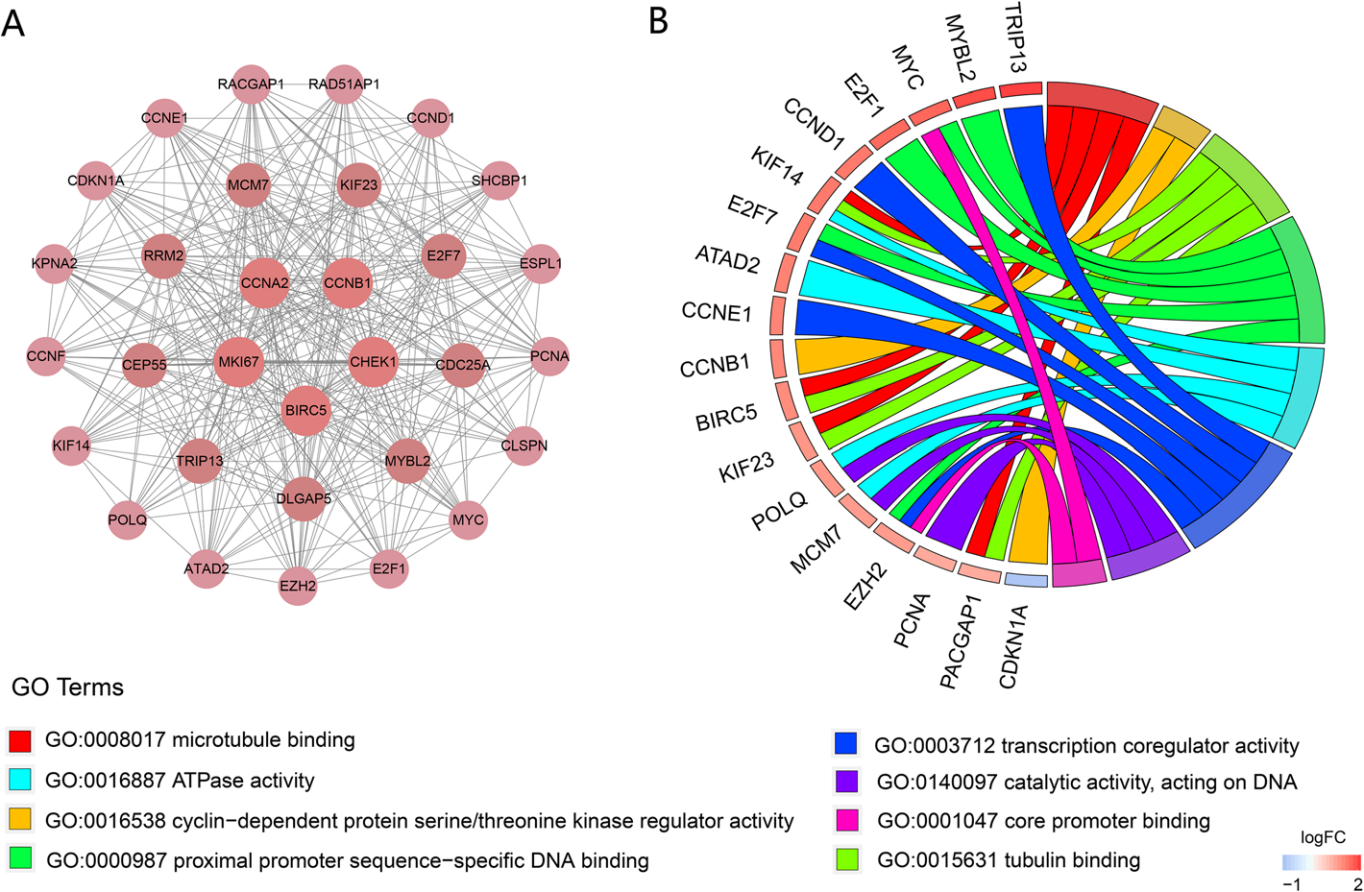
The identification and analysis of the module network. **a** The top modules selected from the PPI network using the plugin MCODE. **b** GO enrichment analysis of genes in the module network. Genes are shown on the left side of the chord diagram and enriched GO terms are shown on the right side. Red bars represent upregulated genes and blue bars represent downregulated genes

The mRNAs in the ceRNA network were used to construct a PPI network based on the STRING database. The PPI network was visualized by Cytoscape and included 265 nodes and 1109 edges (Fig. 4). The cytoHubba plugin was used to identify the top 30 hub genes with the highest degrees (Table 1), of which 7 genes (VEGFA, BIRC5, CDC25A, KPNA2, DLGAP5, CCNE1, and CCNF) were significantly related to OS using Kaplan-Meier analysis (Fig. 5). The MCODE plugin was used in the PPI network to identify the top module, which contained 31 nodes and 341 PPIs (Fig. 6a).

**Table 1:** Top 30 hub genes in the PPI network

mRNA	Degree	Expression	mRNA	Degree	Expression	mRNA	Degree	Expression
MYC	65	up	RRM2	32	up	CCNE1	25	up
VEGFA	54	up	CDC25A	30	up	CEP55	25	up
CCND1	45	up	CDKN1A	30	down	RACGAP1	24	up
EZH2	45	up	PCNA	30	up	ESPL1	24	up
CCNB1	44	up	KPNA2	29	up	RAD51AP1	24	up
CCNA2	42	up	E2F1	29	up	E2F7	24	up
CHEK1	40	up	DLGAP5	28	up	CCNF	23	up
MCM7	37	up	KIF23	28	up	CLSPN	22	up
MKI67	34	up	MYBL2	28	up	KAT2B	20	down
BIRC5	32	up	TRIP13	28	up	MET	20	up

### GO and KEGG enrichment analysis of DEmRNAs in ceRNA

**Fig. 7:**
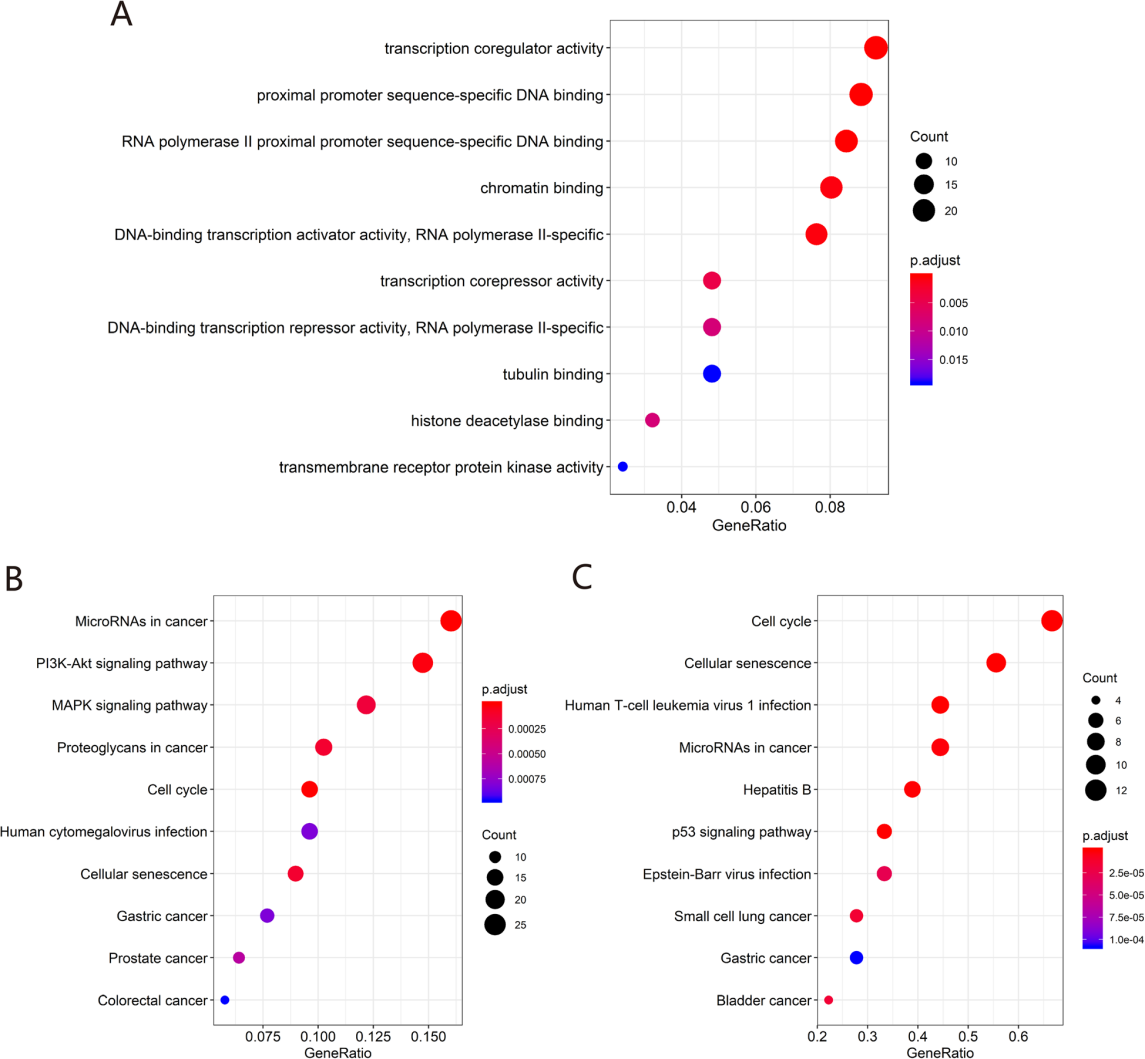
GO and KEGG enrichment analysis of genes in the ceRNA and module networks. **a** GO enrichment analysis of genes in the ceRNA network. **b** KEGG enrichment analysis of genes in the ceRNA network. **c** KEGG enrichment analysis of genes in the module network

We performed GO and KEGG enrichment analysis on 308 mRNAs in the ceRNA network and 31 mRNAs in the module network with the R package “ClusterProfiler”. GO functional enrichment analysis suggested that mRNAs in the ceRNA network were significantly enriched in “proximal promoter sequence-specific DNA binding”, “transcription coregulator activity”, and “RNA polymerase II proximal promoter sequence-specific DNA binding” (Fig. 7a). KEGG pathway analysis showed that ceRNA-related mRNAs were enriched in “microRNAs in cancer”, “cell cycle”, and “PI3K-Akt signaling pathway” (Fig. 7b). Enrichment analysis of mRNAs in the module network showed that the enriched GO terms included “microtubule binding”, “cyclin-dependent protein serine/threonine kinase regulator activity”, and “tubulin binding”, whereas the most enriched KEGG pathways were “cell cycle”, “cellular senescence”, and “p53 signaling pathway” (Fig. 6b and Fig. 7c).

### Clinical feature analysis of key lncRNAs

**Fig. 8:**
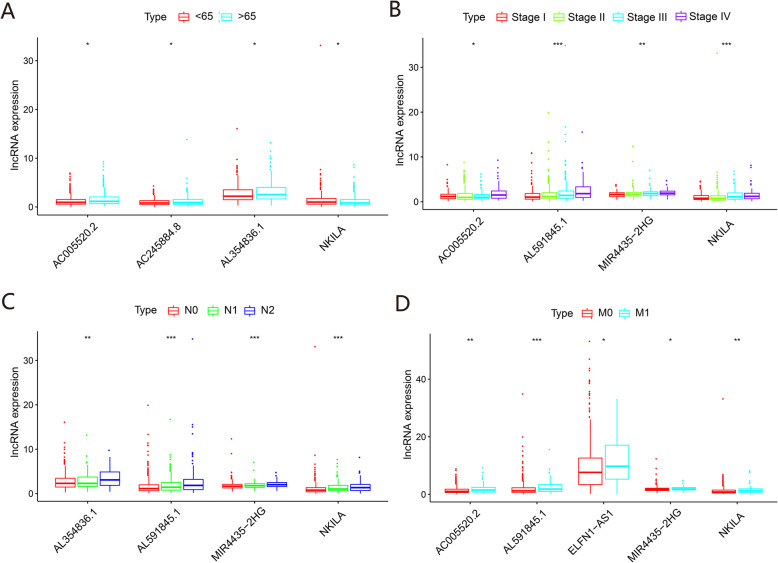
Clinical feature analysis of eleven key lncRNAs. **a** Correlations of lncRNA expression with age. **b** Correlations of lncRNA expression with tumor stage. **c** Correlations of lncRNA expression with pathologic N stage. **d** Correlations of lncRNA expression with pathologic M stage

To further investigate the 11 prognosis-related lncRNAs in the ceRNA network, we analyzed the correlation between lncRNAs and clinical features, including age, sex, tumor stage, and TNM stage. Seven lncRNAs were associated with the clinical features of CRC patients (all *P* < 0.05). Four lncRNAs—NKILA, AC005520.2, AC245884.8, and AL354836.1—were age-related, four lncRNAs—MIR4435-2HG, NKILA, AL591845.1, and AC005520.2—were associated with tumor stage, and six lncRNAs—MIR4435-2HG, ELFN1-AS1, NKILA, AL591845.1, AC005520.2, and AL354836.1—were associated with TNM stage (Fig. 8). The detailed clinical and pathological characteristics of 548 colorectal cancer patients are shown in Table 2.

**Table 2:** Clinicopathological characteristics of 548 colorectal carcinoma patients

Parameter	Subtype	Patients (%)
Age (years)	> 65	311 (56.8%)
	≤65	237 (43.2%)
Gender	Male	292 (53.3%)
	Female	256 (46.7%)
Pathologic stage	Stage I	96 (17.5%)
	Stage II	210 (38.3%)
	Stage III	149 (27.2%)
	Stage IV	78 (14.2%)
	Unknown	15 (2.7%)
Pathologic T	T1	15 (2.7%)
	T2	96 (17.5%)
	T3	373 (68.1%)
	T4	63 (11.5%)
	Tis	1 (0.2%)
Pathologic N	N0	323 (58.9%)
	N1	130 (23.7%)
	N2	94 (17.2%)
	NX	1 (0.2%)
Pathologic M	M0	408 (74.5%)
	M1	77 (14.1%)
	MX	55 (10.0%)
	Unknown	8 (1.5%)

### Co-expression analysis of lncRNAs and mRNAs

**Fig. 9:**
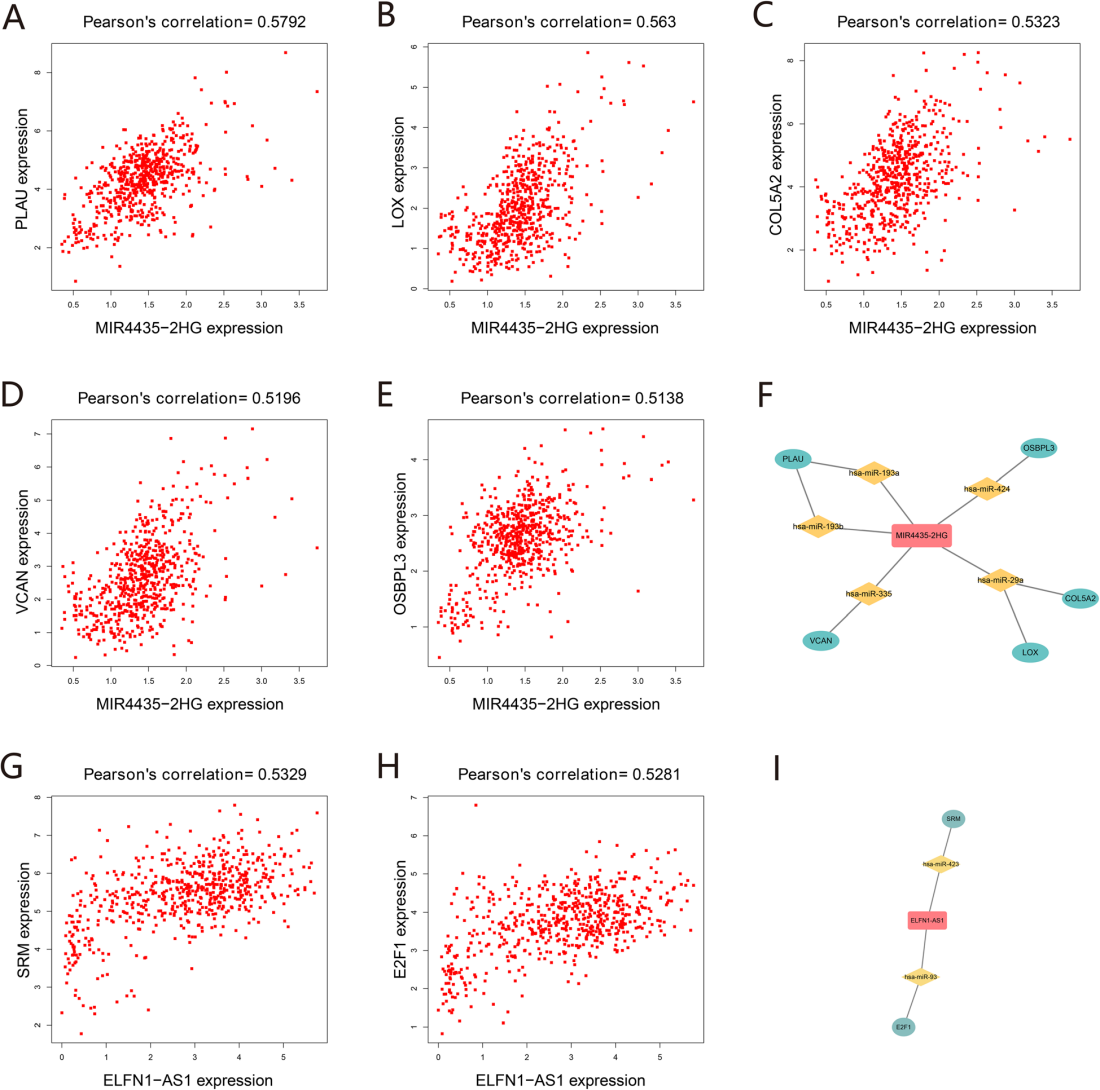
Pearson correlation analysis between lncRNAs and mRNAs. **a**–**e** The co-expression relationship between MIR4435-2HG and paired mRNAs (PLAU, LOX, COL5A1, VCAN and OSBPL3). **f** The ceRNA regulatory subnetwork of MIR4435-2HG. (G–H) The co-expression relationship between ELFN1-AS1 and paired mRNAs (SRM and E2F1). **i** The ceRNA regulatory subnetwork of ELFN1-AS1

To verify the hypothesis that lncRNA positively regulates mRNA expression by interacting with miRNA in the ceRNA network, we performed Pearson correlation analysis between seven clinically relevant lncRNAs and their target mRNAs. The results showed that MIR4435-2HG was positively correlated with 15 mRNAs (r > 0.4, Table 3), of which COL5A2, LOX, OSBPL3, PLAU, and VCAN had a significantly positive correlation with MIR4435-2HG (r > 0.5). We speculated that MIR4435-2HG upregulated the expression of the above five DEmRNAs through different DEmiRNAs (mir-29a, mir-424, mir-335, mir-193a, and mir-193a) (Fig. 9a-f). Our correlation analysis of ELFN1-AS1 showed that seven mRNAs were positively associated with ELFN1-AS1 (r > 0.4, Table 3), whereas SRM and E2F1 had a significantly positive correlation with ELFN1-AS1 (r > 0.5). Based on the ceRNA hypothesis, ELFN1-AS1 may upregulate the expression of SRM and E2F1 by acting as sponges of mir-423 and mir-93, respectively (Fig. 9g-i).

**Table 3:** Positive correlations between lncRNAs and mRNAs

lncRNA	mRNA
MIR4435-2HG	COL5A2, LOX, OSBPL3, PLAU, VCAN, ANGPT2, CBFB, COL3A1, COL4A1, EIF5A2, GPR180, RGS16, SLC7A11, TM4SF18, VMA21
ELFN1-AS1	E2F1, SRM, BIRC5, C20orf27, DUSP2, LMNB2, MYBL2
AL354836.1	FGFRL1
AC245884.8	VEGFA

### Validation of gene expression by GEPIA and immunohistochemistry

**Fig. 10:**
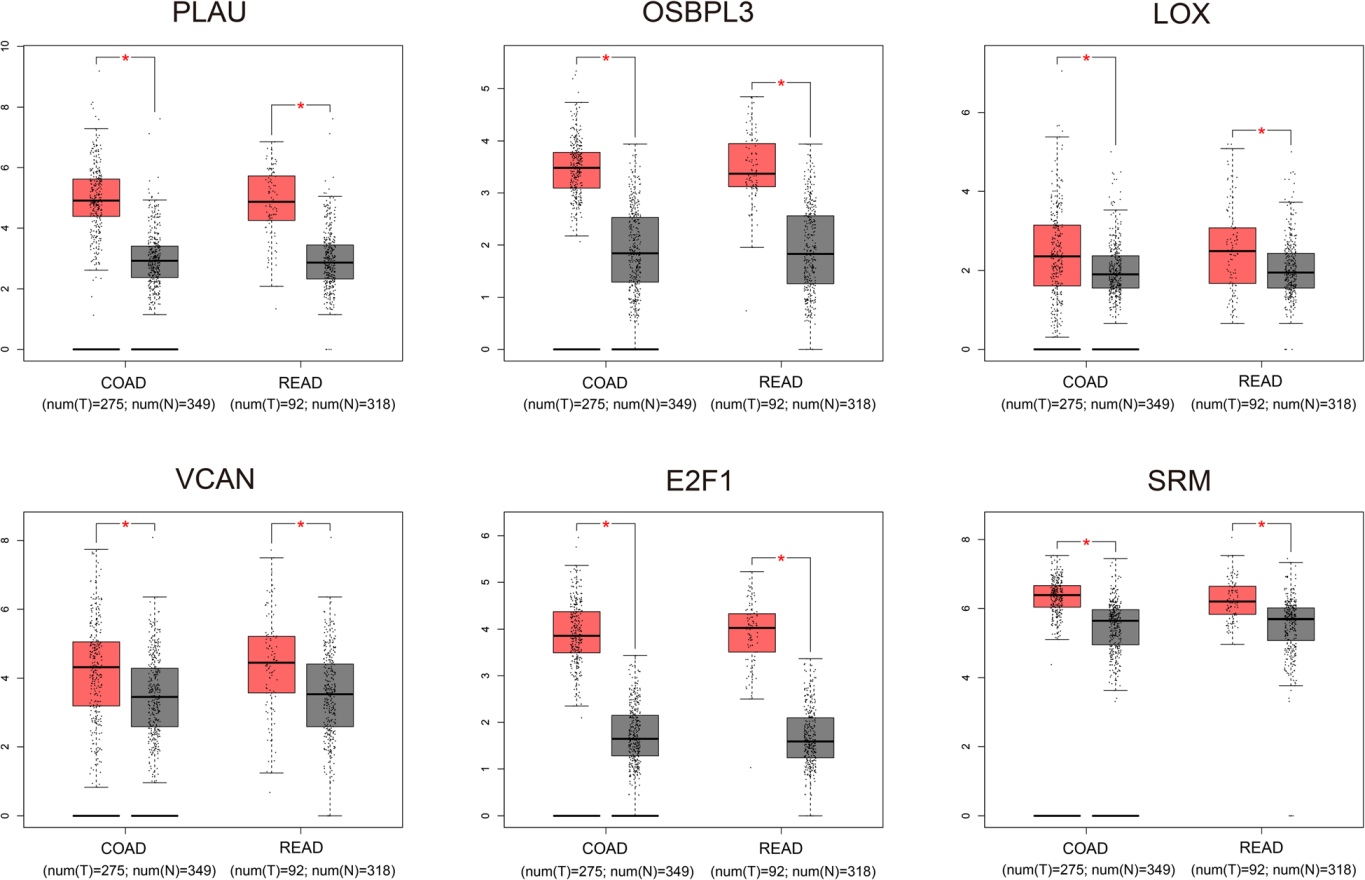
Validation of differential gene expression using Gene Expression Profiling Interactive Analysis (GEPIA). The red bars represent tumor samples, and the gray bars represent normal samples

**Fig. 11:**
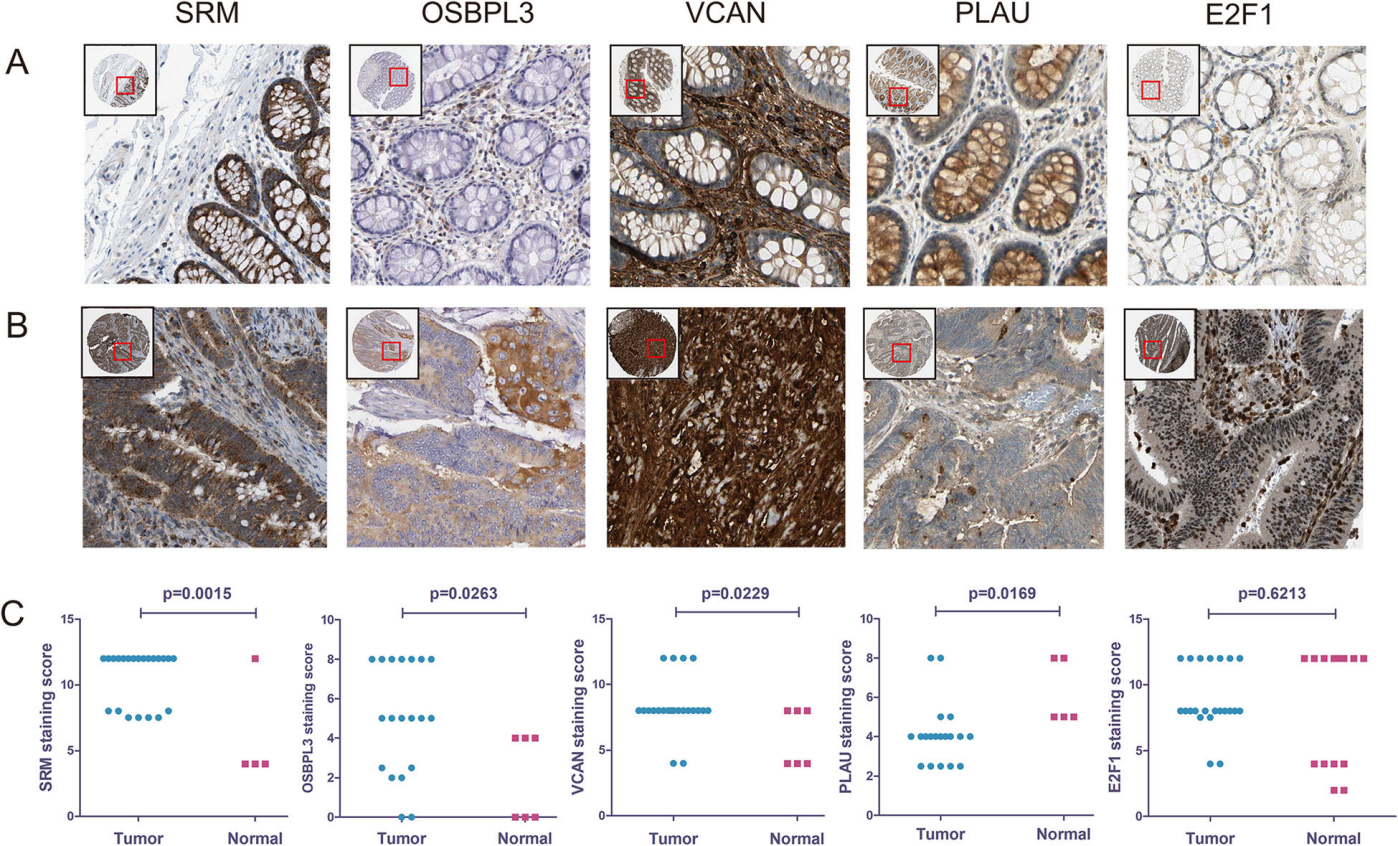
Immunohistochemical analysis of protein expression levels of genes based on The Human Protein Atlas (THPA) database. **a** Immunohistochemical results for normal tissues. **b** Immunohistochemical results for CRC tissues. **c** Plot diagram of staining scores between CRC and normal tissues

We used TCGA and GTEx datasets in GEPIA to further validate the differential expression of genes between tumor and normal samples. The expression levels of PLAU, OSBPL3, LOX, VCAN, E2F1, and SRM were upregulated in COAD and READ tissues compared with normal tissues (all *P* < 0.05, Fig. 10). However, the expression of COL5A2 showed no differential expression between tumor and normal tissues. Immunohistochemistry results were obtained from the THPA database, and the staining scores were quantified to verify the protein expression levels of the genes. The staining scores for SRM (*P* = 0.0015), OSBPL3 (*P* = 0.0263), and VCAN (*P* = 0.0229) were higher in the CRC tissue than normal tissue, whereas PLAU (*P* = 0.0169) showed the opposite pattern. There was no significant difference between the E2F1 staining scores (*P* = 0.6213) for CRC and normal tissue (Fig. 11).

### GSEA-predicted biological functions of genes

**Fig. 12:**
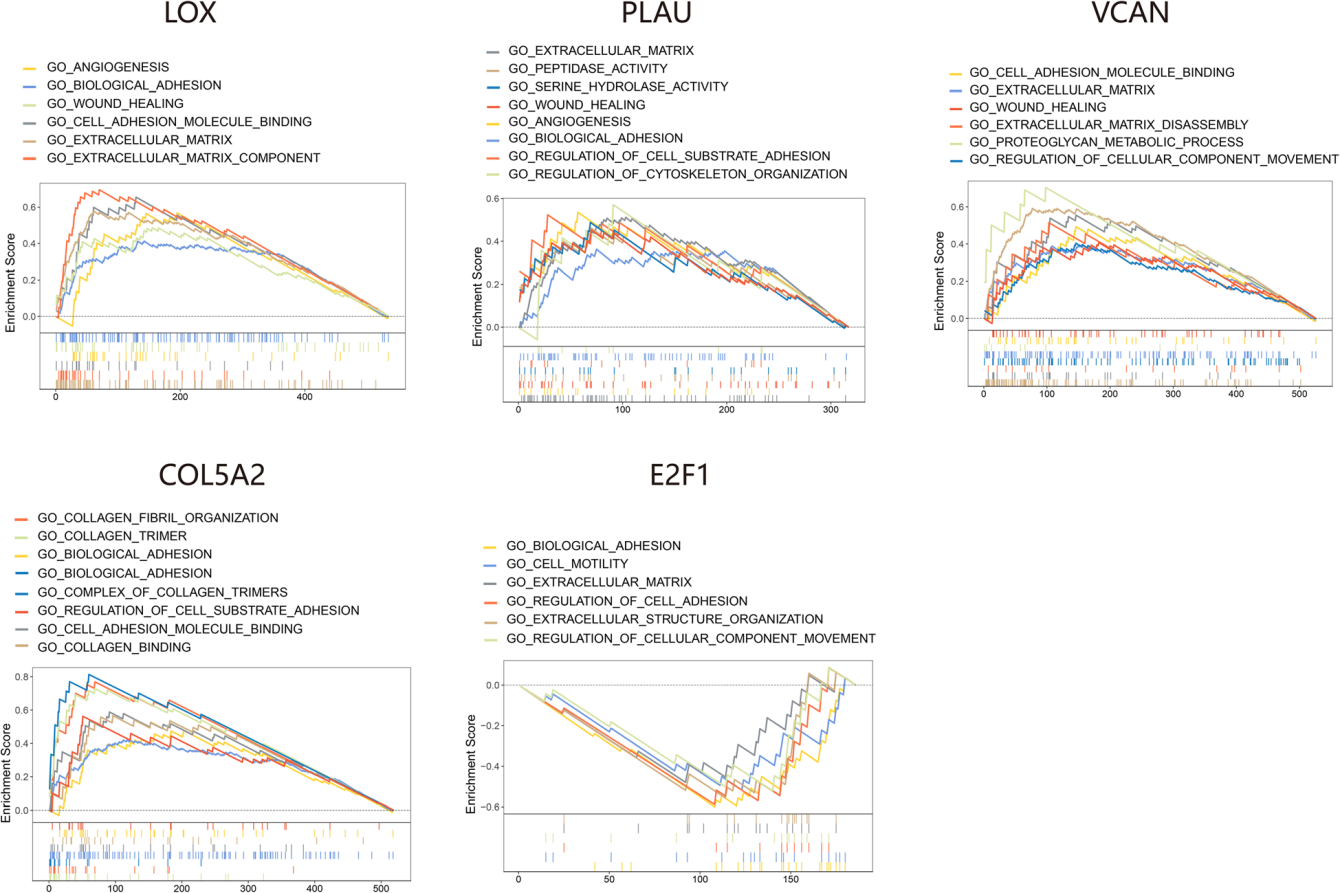
Gene set enrichment analysis (GSEA) revealed enriched KEGG pathways based on gene expression levels

We used GSEA analysis to predict the enriched KEGG pathways between high and low expressions of genes in a subnetwork. PLAU, encoding a secreted serine protease, was positively correlated with “extracellular matrix”, “angiogenesis”, and “serine hydrolase activity”. LOX was significantly enriched in “extracellular matrix”, “cell adhesion molecule binding”, and “angiogenesis”. VCAN belongs to the aggrecan/versican proteoglycan family, which was positively correlated with “extracellular matrix component”, “cell adhesion molecule binding”, and “proteoglycan metabolic process”. COL5A2 encodes one alpha chain of fibrillar collagen trimers. The high expression of COL5A2 was correlated with “extracellular matrix”, “collagen trimer”, and “collagen fibril organization”, whereas E2F1 was negatively correlated with “biological adhesion”, “regulation of cell adhesion”, and “cell motility” (Fig. 12).

### Tumor-infiltrating immune cells in CRC

**Fig. 13:**
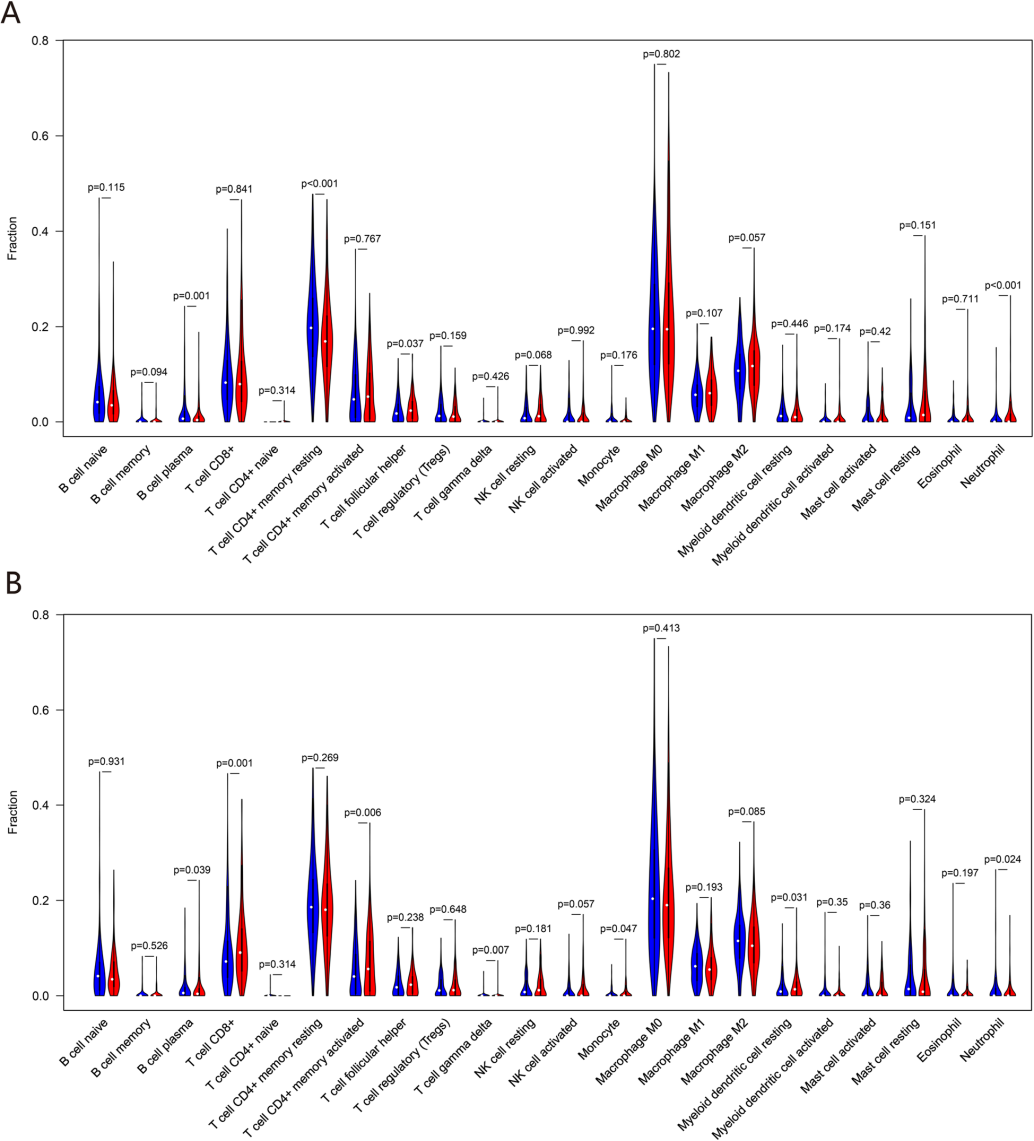
Violin plots for tumor-infiltrating immune cells. **a** Comparison of immune cell proportions between high and low MIR4435-2HG expression groups. **b** Comparison of immune cell proportions between high and low ELFN1-AS1 expression groups. The blue bars represent the low expression groups, and the red bars represent the high expression groups

A cohort of 568 CRC patients was divided into high and low expression groups based on lncRNA expression. The results of the Wilcoxon rank-sum test revealed that plasma B cells (*P* = 0.001) and CD4+ memory resting T cells (*P* < 0.001) were greater in patients with low MIR4435-2HG expression, while follicular helper T cells (*P* = 0.037) and neutrophils (*P* < 0.001) were greater in patients with high MIR4435-2HG expression (Fig. 13a). The high ELFN1-AS1 expression group had significantly higher numbers of plasma B cells (*P* = 0.039), CD4+ memory-activated T cells (*P* = 0.006), gamma delta T cells (*P* = 0.007), monocytes (*P* = 0.047), and resting myeloid dendritic cells (*P* = 0.031). The low ELFN1-AS1 expression group had higher numbers of CD8+ T cells (*P* = 0.001) and neutrophils (*P* = 0.024) (Fig. 13b). Correlation analysis was performed to determine the co-expression patterns among 22 tumor-infiltrating immune cell types (Supplementary Figure S4).

## Discussion

Colorectal cancer (CRC) is one of the leading causes of cancer-related death worldwide. Increasing evidence has shown that dysregulated lncRNAs or lncRNA-mediated ceRNA networks are involved in the progression and prognosis of the CRC. LncRNAs suppress miRNA-induced translational inhibition of gene expression by competitively binding to miRNA response elements. In recent years, they have gained wide attention due to their important roles in tumor progression, patient prognosis, and immune infiltration [7, 13, 23]. The lncRNA H19/miR-29b-3p/PGRN axis promotes EMT in CRC and counts a great deal for seeking appropriate diagnostic biomarkers and therapeutic targets for CRC [11]. The oncogenic HOTAIR/miR-331-3p/HER2 axis is associated with tumor progression and poor prognosis in gastric cancer [24]. In addition, the RP11-1094 M14.8/miR-1269a/CXCL9 axis is a potential prognostic marker for gastric cancer with different degrees of immune cell infiltration [13]. Thus, identifying lncRNAs and their associated ceRNA networks as prognostic factors may provide a foundation for the development of early diagnostic biomarkers and therapeutic targets in CRC.

In this study, we analyzed TCGA sequence data from CRC patients and constructed a prognostic ceRNA network to explore the potential function of lncRNAs and their related genes. MIR4435-2HG and ELFN1-AS1 were identified as potential prognostic biomarkers and therapeutic targets in CRC based on survival and clinical feature analysis. MIR4435-2HG promotes CRC growth and metastasis through the miR-206/YAP1 axis and predicts poor prognosis in CRC patients [25–27]. ELFN1-AS1 is elevated in CRC tissues, and its upregulation promotes CRC cell proliferation and migration and inhibits apoptosis by competing with the endogenous miR-4644/TRIM44 axis [28, 29]. While downregulation of ELFN1-AS1 suppresses the activation of extracellular signal-regulated protein kinase (ERK), decreases vimentin expression, and increases E-cadherin expression.

Based on these ceRNA networks and lncRNA-mRNA co-expression relationships, we obtained two novel ceRNA regulatory subnetworks for MIR4435-2HG and ELFN1-AS1. According to the univariate and multivariate Cox analysis, ELFN1-AS1 was identified as an independent prognostic factor for CRC patients. MIR4435-2HG positively regulated PLAU, VCAN, LOX, COL5A2, and OSBPL3 mRNA expression by acting as a miRNA sponge. PLAU upregulation in primary tumor tissues is positively correlated with distant metastasis and indicates poor patient prognosis in CRC [30]. MiR-193a inhibits CRC cell growth and progression partly by downregulating the expression of target gene PLAU, which is consistent with our prediction of PLAU/miR-193a interaction [31]. VCAN is overexpressed in various human malignancies, including CRC [32–37], and CRC patients with high VCAN expression have worse disease outcomes than those with lower expression [38]. Upregulation of LOX expression is correlated with the progression and metastasis of various solid tumors [39]. There have been contradictory reports regarding the role of LOX in CRC. Csiszar et al. [40] reported that reduced LOX expression in colon tumor tissue supports the function of LOX as a tumor suppressor gene. However, recent studies found that LOX expression was significantly increased in tumor tissue and correlated with poor clinical outcomes of CRC patients [41, 42]. COL5A2 is upregulated in CRC tissue compared to normal tissue, suggesting that COL5A2 is involved in CRC carcinogenesis [43]; however, there are no reports about the role of OSBPL3 in CRC. E2F1 and SRM were predicted to be co-expressed genes involved in the ELFN1-AS1 regulatory network. Several studies have demonstrated that E2F1 is an oncogene involved in CRC proliferation and metastasis. Higher E2F1 expression levels indicate a shorter OS in CRC patients [44, 45]. However, there is also evidence to support E2F1 as a tumor suppressor gene involved in the induction of apoptosis in CRC [46, 47]. SRM encodes one of the key enzymes in the polyamine biosynthesis pathway. Polyamines are essential for cell proliferation and growth, and high expression of polyamines has been linked to several tumors, including CRC [48–52]. However, the direct correlation between the expression of SRM and human tumorigenesis remains unclear.

Subsequently, we performed GSEA analysis to explore the enriched biological pathways of the co-expressed genes. Most were enriched in the “extracellular matrix component,” “angiogenesis,” and “cell adhesion molecule binding,” which correlated with cancer pathogenesis. The extracellular matrix (ECM) is the main component of the tumor microenvironment and is composed of proteins, glycoproteins, proteoglycans, and polysaccharides [53]. Disorders in the ECM structure and composition promote cellular transformation and metastasis during cancer progression [54]. Angiogenesis is the formation of new blood vessels from existing vasculature, an important event in tumor growth, invasion into surrounding tissue, and distant metastasis [55]. Inhibition of angiogenesis has been a valuable therapeutic strategy for many solid tumors. Cell adhesion molecules (CAMs) play an important role in the interaction of cells with other cells and their surrounding intercellular environment. The role of CAMs in cancer involves angiogenesis, disruption of tissue architecture, and loss of contact with other cells and the ECM [56, 57].

Tumor-infiltrating immune cells (TIICs) include T cells, dendritic cells (DCs), B cells, macrophages, neutrophils, mast cells, and natural killer (NK) cells. They are major components of the complex microenvironment that regulates tumor progression and predicts prognosis in CRC [58–60]. To further explore the immune infiltration-related mechanism of how ceRNA regulates CRC prognosis, the estimated proportions of 22 tumor-infiltrating immune cell types in CRC were evaluated by CIBERSORT analysis based on MIR4435-2HG and ELFN1-AS1 expression levels. Our results revealed that nine immune cell types, including CD4+ memory resting T cells, CD4+ memory activated T cells, CD8+ T cells, gamma delta T cells, follicular helper T cells, plasma B cells, neutrophils, monocytes, and resting myeloid DCs, showed statistical differences between the high and low expression groups. Several recent studies reported that the type, density, and location of tumor-infiltrating immune cells are associated with favorable outcomes in CRC [58, 61]. The densities of CD4+ and CD8 + memory T cells were negatively correlated with local tumor invasion based on the T stage of the TNM classification [59]. DCs are potent antigen-presenting cells that play an important role in initiating, programming, and regulating antitumor immune responses. DC-based immunotherapy has been used as anti-cancer immunotherapy to treat various cancers, including CRC [62, 63]. The peripheral neutrophil-to-lymphocyte ratio (NLR) and lymphocyte-to-monocyte ratio (LMR) are prognostic factors for CRC patients [64, 65]. The association of MIR4435-2HG and ELFN1-AS1 lncRNA expression with tumor-infiltrating immune cell types supports their role as valuable prognostic markers and immunotherapeutic targets in CRC.

Nevertheless, there are still some limitations to the present study. First, the data used in this study were mainly from Western countries and lacked data from the Asian population. Second, the number of normal cases was relatively small, and a larger number of paired samples are needed for further analysis. Third, the multiple sources of the online verification data need to be further confirmed by homogeneous research samples and systematic experimental studies. For example, although the immunohistochemistry data were extracted from The Human Protein Atlas (THPA) to verify the upregulation of the identified genes in CRC at the protein level, the exact molecular mechanisms involving these hub genes have not been experimentally investigated in the present study. In addition, we observed opposing PLAU expression patterns at the mRNA and protein levels (Fig. 10 and Fig. 11), which is inconsistent with previous reports [66, 67]. Taken together, further experimental studies based on a larger sample size are needed to verify our hypotheses.

## Conclusions

The regulatory networks of MIR4435-2HG and ELFN1-AS1 are considered to be closely associated with CRC prognosis. Although further functional experiments are required, our findings contribute to the development of MIR4435-2HG and ELFN1-AS1 as potential prognostic biomarkers and therapeutic targets in CRC.

## Supplementary Information

**Additional file 1.** Clustered heatmaps of differentially expressed RNAs. (A) Heatmaps of lncRNAs. (B) Heatmaps of miRNAs. (C) Heatmaps of mRNAs.

**Additional file 2.** Construction of a survival-related ceRNA network. Red nodes represent the upregulated RNAs, and blue nodes represent the downregulated RNAs.

**Additional file 3.** Univariate and multivariable Cox regression analysis of RNAs involved in the ceRNA network.

**Additional file 4.** Correlation of 22 tumor-infiltrating immune cell types.

**Additional file 5.** Certificate of English copyediting service.

## Data Availability

The data information of this study was obtained from the Cancer Genome Atlas (TCGA; http://cancergenome.nih.gov/), the Human Protein Atlas (THPA; https://www.proteinatlas.org) and the GTEx projects.

## Abbreviations

lncRNA: Long noncoding RNA
miRNA: MicroRNA
mRNA: Message RNA
ceRNA: Competing endogenous RNA
TCGA: The Cancer Genome Atlas
DElncRNA: Differentially expressed lncRNA
DEmiRNA: Differentially expressed miRNA
DEmRNA: Differentially expressed mRNA
GEPIA: Gene Expression Profiling Interactive Analysis
GSEA: Gene Set Enrichment Analysis
CRC: Colorectal cancer
OS: Overall survival
EMT: Epithelial-to-mesenchymal transition
PPI: Protein–protein interaction
GO: Gene Ontology
KEGG: Kyoto Encyclopedia of Genes and Genomes
STRING: Search Tool for the Retrieval of Interacting Genes
GTEx: Genotype-Tissue Expression
THPA: The Human Protein Atlas
NES: Normalized enrichment score
COAD: Colon adenocarcinoma
READ: Rectal adenocarcinoma
ERK: Extracellular-signal regulated protein kinase
