# Supplementary material for: Comprehensive analysis of ceRNA networks reveals prognostic lncRNAs related to immune infiltration in colorectal cancer
Source: BMC Cancer. 2021 Mar 9;21:255. doi: 10.1186/s12885-021-07995-2 (PMC7941714; doi:10.1186/s12885-021-07995-2)
Supplement: Supplementary file 1 — Additional file 1. Clustered heatmaps of differentially expressed RNAs. (A) Heatmaps of lncRNAs. (B) Heatmaps of miRNAs. (C) Heatmaps of mRNAs. [file 12885_2021_7995_MOESM1_ESM.docx]

**Additional file 1.** Clustered heatmaps of differentially expressed RNAs. (A) Heatmaps of lncRNAs. (B) Heatmaps of miRNAs. (C) Heatmaps of mRNAs.

**
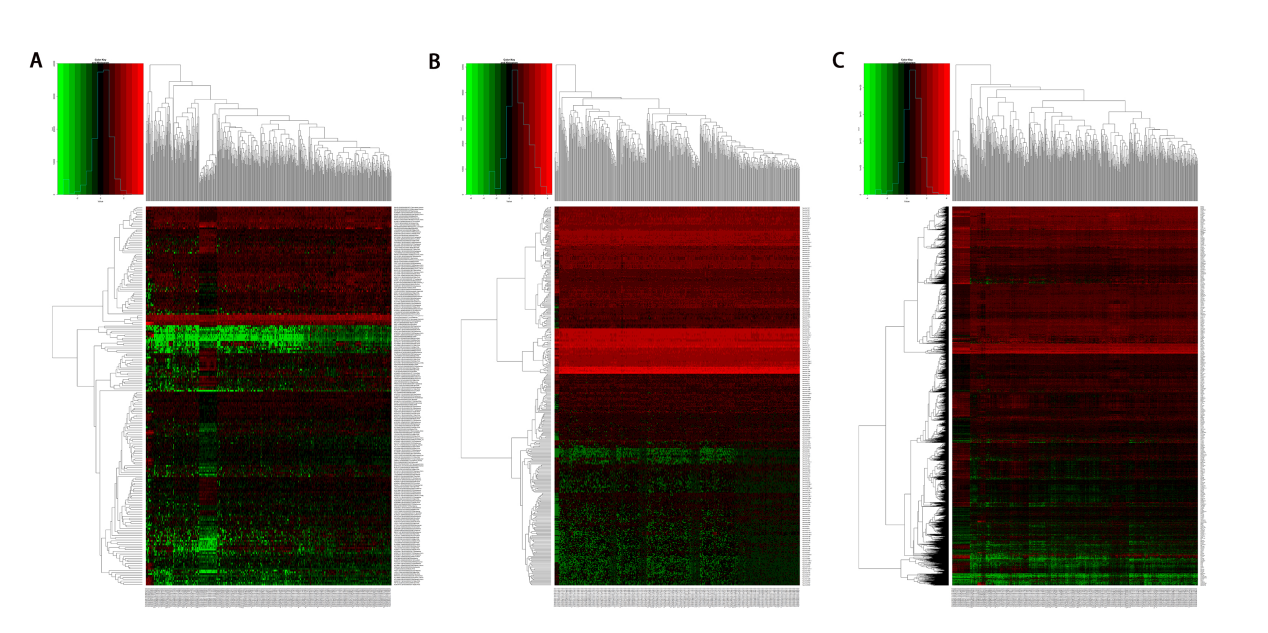
**
