# Supplementary material for: Comprehensive analysis of ceRNA networks reveals prognostic lncRNAs related to immune infiltration in colorectal cancer
Source: BMC Cancer. 2021 Mar 9;21:255. doi: 10.1186/s12885-021-07995-2 (PMC7941714; doi:10.1186/s12885-021-07995-2)

**Additional file 2.** Construction of a survival-related ceRNA network. Red nodes represent the upregulated RNAs and blue nodes represent the downregulated RNAs.


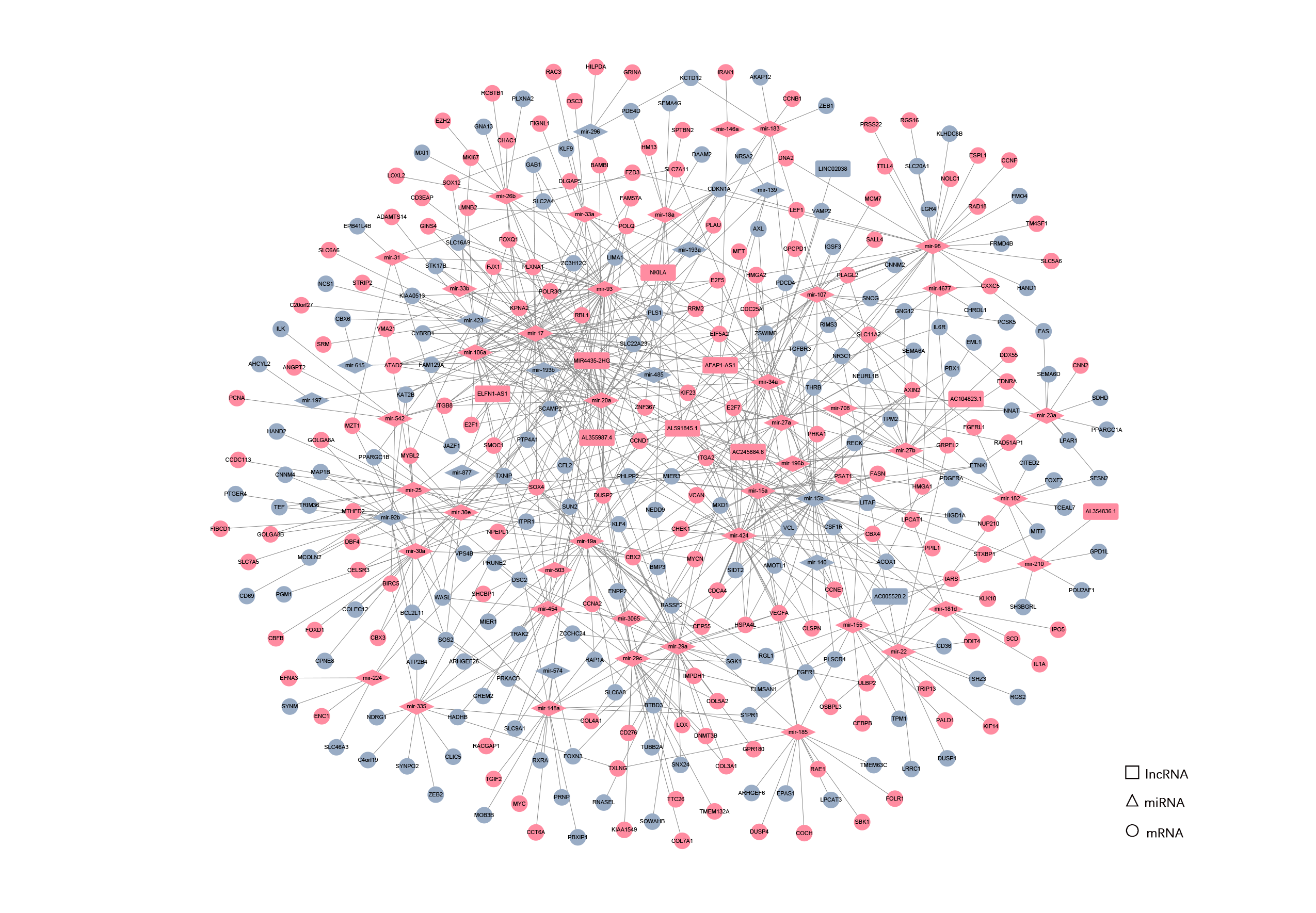

Supplement: Supplementary file 2 — Additional file 2. Construction of a survival-related ceRNA network. Red nodes represent the upregulated RNAs, and blue nodes represent the downregulated RNAs. [file 12885_2021_7995_MOESM2_ESM.docx]
