# Supplementary material for: Comprehensive analysis of ceRNA networks reveals prognostic lncRNAs related to immune infiltration in colorectal cancer
Source: BMC Cancer. 2021 Mar 9;21:255. doi: 10.1186/s12885-021-07995-2 (PMC7941714; doi:10.1186/s12885-021-07995-2)
Supplement: Supplementary file 3 — Additional file 3. Univariate and multivariable Cox regression analysis of RNAs involved in the ceRNA network. [file 12885_2021_7995_MOESM3_ESM.docx]

**Additional file 3.** Univariate and multivariable Cox regression analysis of RNAs involved in the ceRNA network.

| **Variable** | **Univariate analysis** | | **Multivariate analysis** | |
| --- | --- | --- | --- | --- |
|  | **HR(95%CI)** | **P-value** | **HR(95%CI)** | **P-value** |
| DElncRNA | | | | |
| ELFN1-AS1 | 1.23(1.04-1.47) | 0.01892873 | 1.25(1.01-1.56) | 0.043330126* |
| AC005520.2 | 1.42(1.05-1.91) | 0.02127087 | 1.38(0.97-1.97) | 0.075612787 |
| AL354836.1 | 1.35(1.00-1.83) | 0.04953354 | 0.95(0.58-1.56) | 0.836967997 |
| MIR4435-2HG | 1.81(1.09-3.02) | 0.0228869 | 1.32(0.59-2.95) | 0.504493176 |
| AL591845.1 | 1.28(1.04-1.57) | 0.01836881 | 1.08(0.80-1.46) | 0.633619431 |
| NKILA | 1.58(1.20-2.07) | 0.00105998 | 1.30(0.85-1.99) | 0.230296196 |
| DEmRNA | | | | |
| FOXD1 | 1.34(1.09-1.65) | 0.00489383 | 1.02(0.77-1.34) | 0.910636053 |
| HAND1 | 1.36(1.03-1.80) | 0.02972407 | 1.51(1.00-2.29) | 0.052176287 |
| NCS1 | 1.41(1.02-1.94) | 0.03775377 | 1.00(0.62-1.63) | 0.985293953 |
| ACOX1 | 0.57(0.37-0.86) | 0.00804259 | 0.55(0.30-1.01) | 0.054406083 |
| SPTBN2 | 1.37(1.01-1.85) | 0.04161665 | 1.51(1.02-2.24) | 0.041189148* |
| CCNB1 | 0.69(0.51-0.93) | 0.01523988 | 1.23(0.64-2.39) | 0.532739336 |
| RIMS3 | 0.63(0.40-0.97) | 0.03492597 | 0.72(0.42-1.26) | 0.253116636 |
| TUBB2A | 1.37(1.03-1.82) | 0.03187147 | 1.44(0.97-2.14) | 0.073197003 |
| PPARGC1A | 0.66(0.46-0.94) | 0.02284588 | 0.69(0.44-1.08) | 0.108159982 |
| OSBPL3 | 1.49(1.02-2.17) | 0.0373454 | 1.06(0.66-1.70) | 0.818828263 |
| KPNA2 | 0.57(0.39-0.84) | 0.0040458 | 0.51(0.24-1.07) | 0.07507942 |
| CD36 | 1.42(1.06-1.92) | 0.01982018 | 1.33(0.84-2.11) | 0.216288474 |
| CCNF | 0.50(0.34-0.76) | 0.00103036 | 0.39(0.21-0.72) | 0.002573394** |
| PBXIP1 | 1.55(1.10-2.16) | 0.01124881 | 1.24(0.67-2.28) | 0.493704176 |
| MOB3B | 0.70(0.50-0.99) | 0.04550973 | 1.12(0.62-2.02) | 0.695989481 |
| JAZF1 | 1.56(1.06-2.30) | 0.02392013 | 0.98(0.44-2.19) | 0.965198223 |
| CCNA2 | 0.72(0.53-0.97) | 0.03331891 | 1.21(0.60-2.45) | 0.596766483 |
| SNCG | 1.32(1.04-1.67) | 0.0220472 | 0.83(0.56-1.21) | 0.329875126 |
| BIRC5 | 0.67(0.48-0.93) | 0.01584712 | 1.14(0.56-2.31) | 0.726262774 |
| CXXC5 | 0.66(0.50-0.87) | 0.00377092 | 0.72(0.47-1.08) | 0.114995953 |
| TPM2 | 1.31(1.09-1.57) | 0.00433317 | 0.98(0.68-1.40) | 0.896109354 |
| FJX1 | 1.65(1.21-2.25) | 0.00160568 | 1.38(0.89-2.15) | 0.147444296 |
| PCSK5 | 1.52(1.09-2.12) | 0.01241736 | 1.37(0.87-2.16) | 0.171216271 |
| VEGFA | 1.40(1.02-1.93) | 0.03708641 | 1.64(1.05-2.55) | 0.029558772* |
| CHRDL1 | 1.22(1.02-1.47) | 0.03256092 | 0.80(0.56-1.15) | 0.233819048 |
| RGS16 | 1.28(1.02-1.61) | 0.03259427 | 0.86(0.62-1.20) | 0.377679416 |
| SALL4 | 1.38(1.03-1.86) | 0.03258171 | 0.84(0.52-1.36) | 0.481391172 |
| C4orf19 | 0.65(0.49-0.87) | 0.00367243 | 0.96(0.61-1.53) | 0.878428289 |
