# Supplementary figures and images for: Comprehensive analysis of ceRNA networks reveals prognostic lncRNAs related to immune infiltration in colorectal cancer
Source: BMC Cancer. 2021 Mar 9;21:255. doi: 10.1186/s12885-021-07995-2 (PMC7941714; doi:10.1186/s12885-021-07995-2)

**Additional file 4.** Correlation of 22 tumor-infiltrating immune cell types.


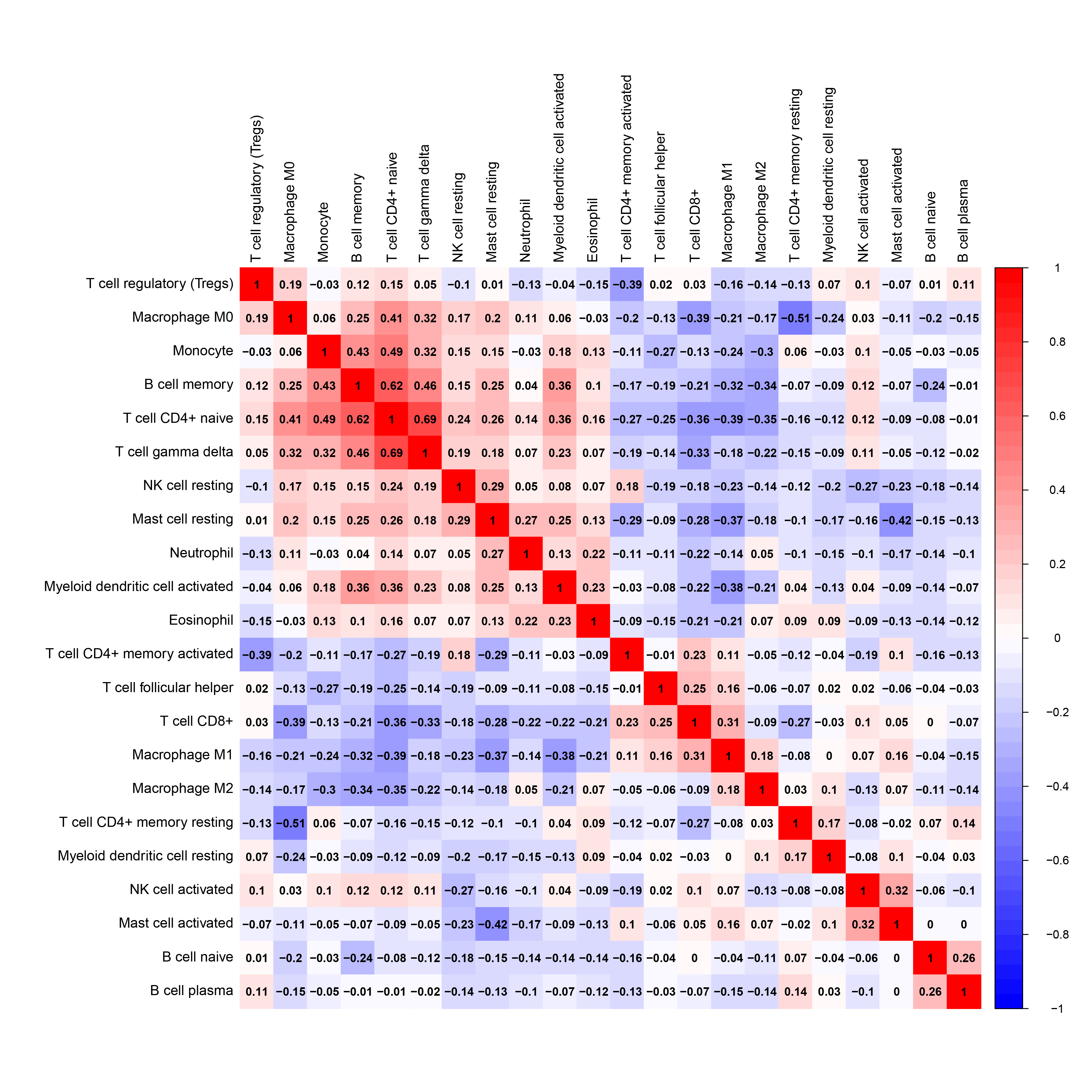

Supplement: Supplementary file 4 — Additional file 4. Correlation of 22 tumor-infiltrating immune cell types. [file 12885_2021_7995_MOESM4_ESM.docx]

**Additional file 5.** Certificate for English copyediting service.

**
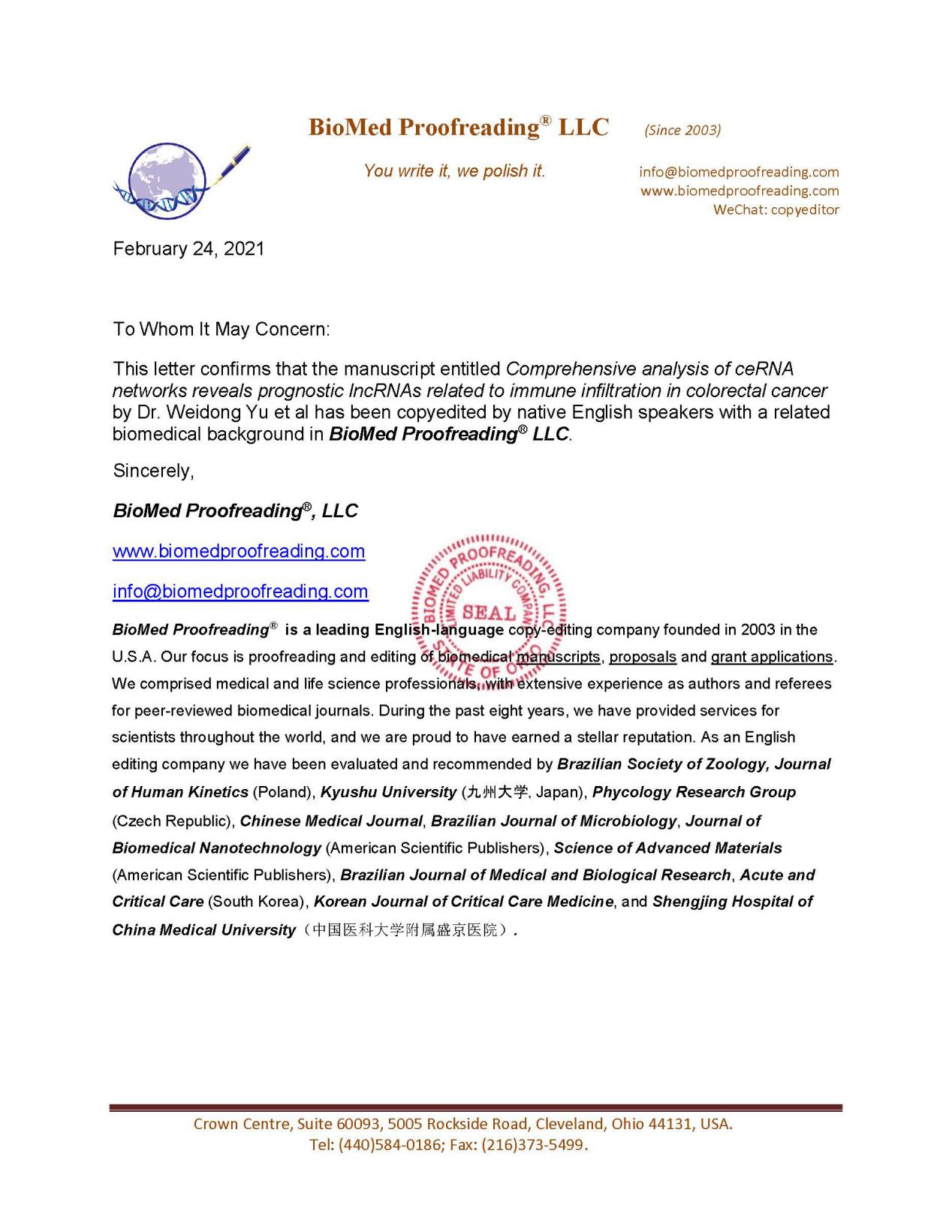
**

Supplement: Supplementary file 5 — Additional file 5. Certificate of English copyediting service. [file 12885_2021_7995_MOESM5_ESM.docx]
